# Detecting Perceived Unfair Treatment Among US College Students Using Mobile Sensing: Pilot Machine Learning Study

**DOI:** 10.2196/78657

**Published:** 2025-10-31

**Authors:** Yiyi Ren, Raghu Mulukutla, Jennifer Mankoff, Anind K Dey

**Affiliations:** 1 Information School University of Washington Seattle, WA United States; 2 College of Computing Georgia Institute of Technology Atlanta, GA United States; 3 Paul G. Allen School of Computer Science & Engineering University of Washington Seattle, WA United States

**Keywords:** passive sensing, perceived discrimination, anomaly detection, digital phenotyping, mental health, mobile health

## Abstract

**Background:**

Experiences of unfair treatment on college campuses are linked to adverse mental and physical health outcomes, highlighting the need for interventions. However, detecting such experiences relies mainly on self-reports. No prior research has examined the feasibility of using mobile sensing via smartphones and wearables for the passive detection of these experiences.

**Objective:**

This pilot study explores the potential of using passive sensing to detect daily experiences of perceived unfair treatment (PUT) after they occur. It aims to develop and evaluate machine learning models against naive baselines and establish a benchmark for future research.

**Methods:**

We analyzed data from 201 undergraduate students collected over two 10-week academic terms in 2018. PUT was self-reported at the daily level via ecological momentary assessment (EMA) surveys, with 413 of 9629 (4.3%) total responses indicating unfair treatment. We implemented two modeling approaches with distinct training schemes: (1) supervised classification models trained in a user-independent manner using data from different individuals, and (2) anomaly detection models trained in a user-dependent manner using historical data from the same individuals. Classification performance was assessed using stratified group 5-fold cross-validation for user-independent models and a chronological train-test split for user-dependent models.

**Results:**

Of the 201 study participants, 110 reported experiencing unfair treatment at least once. On average, participants reported unfair treatment in 4.66% of their EMA responses (95% CI 3.13% to 6.19%). User-independent classification models showed mixed performance (AUC-ROC [area under the receiver operating characteristic curve]: 0.546-0.640, AUC-PR [area under the precision-recall curve]: 0.047-0.093, *F*_1_-score: 0.070-0.121). Tree-based models, particularly light gradient boosting machine (LightGBM) and Random Forest, outperformed all 3 baselines in AUC-ROC and AUC-PR; LightGBM also improved the *F*_1_-score. In comparison, user-dependent anomaly detection models performed better, with the multiday long short-term memory-AE model (50 features, 7-day window) achieving the highest recall (0.830, +73.3%, *P*<.001) and *F*_1_-score (0.391, +24.9%, *P*<.001) without reducing precision (0.256), and improving AUC-PR by 45.9% and AUC-ROC by 21.6% relative to naive baselines (*P*<.001). Feature importance analysis identified key behavioral patterns for population-level detection, including increased time spent off campus, elevated evening and nighttime activity, reduced indoor mobility on campus, prolonged screen use, delayed sleep onset, and shorter sleep duration.

**Conclusions:**

Mobile sensing shows promise for detecting daily experiences of PUT in college students and identifying associated behavioral patterns. Our findings highlight opportunities for timely interventions through mobile technology to mitigate the impact of these experiences on students’ mental health and well-being.

## Introduction

Unfair treatment refers to the act of denying individuals equal and just consideration based on characteristics such as race, gender, age, or disability [1]. In US college environments, perceived unfair treatment (PUT) remains a persistent issue with significant impacts on students' lives [2-5]. While the literature often uses discrimination interchangeably with unfair treatment [6,7], our study adopts the broader construct of PUT, which includes not only overt acts of discrimination but also subtle, everyday indignities known as microaggressions [8]. Drawing on past research on perceived discrimination to contextualize our work, we define PUT as an individual’s subjective perception of being treated unjustly based on group characteristics. Within the university setting, this can manifest in various ways. For example, students being stereotyped by faculty, perceiving bias in academic evaluation, being unfairly blamed for dorm noise, or encountering classmates who express surprise at a minority student’s success [9].

These experiences of PUT can induce acute physiological and emotional distress [10-13], contribute to increased suicidality [14-16], substance use [3,17,18], poor academic performance [19,20], and have long-lasting effects on social well-being and mental health, such as disrupted personality development [21], hindered career growth [22,23], eating disorders [24,25], and impaired social integration [26,27]. Despite their prevalence, many incidents go unreported [28-30], resulting in limited institutional awareness and response. Developing reliable methods to detect these experiences soon after they occur is crucial for enabling timely interventions [31,32] and providing social support [33,34] for at-risk students.

Currently, PUT is primarily studied and detected based on self-reports, either via standard questionnaires such as the Major Experiences of Discrimination and the Everyday Discrimination Scale [35-37], or through the Experience Sampling Method, also known as Ecological Momentary Assessment (EMA) [38-40]. While these self-reported measures provide valuable insights into individuals’ experiences, they are subject to recall and nonresponse biases, inconsistent reporting, and significant participant burden [41-43], making them challenging to scale for continuous or longitudinal monitoring and detection. To the best of our knowledge, no framework or system exists that can automatically or passively detect PUT after it happens.

The health care landscape is undergoing a notable transformation, shifting towards noninvasive and accessible methods for early detection [44-46]. This shift is being largely fueled by advancements in mobile sensing technology and the growing interest in machine learning, which together offer unprecedented opportunities [47-50]. Numerous studies have highlighted the effectiveness of these technologies in addressing mental health and well-being tasks, such as depression screening and detection [51-55]. Concurrently, emerging research has begun to uncover short-term behavioral correlates of discrimination experiences [39,56,57], including changes in physical activity, sleep patterns, phone use, and social interactions—behaviors that can be objectively measured through smartphone and wearable sensors. However, most previous studies have focused on uncovering health and behavioral associations with perceived discrimination. While these analytical approaches provide valuable insights, they do not directly address the challenge of detection. Therefore, this study aims to fill this gap by developing and evaluating models that detect PUT after it occurs based on behavioral changes. To our knowledge, this is the first work to explore passive detection using mobile sensing data. Our goal is to establish a benchmark that can inform and advance future research in this emerging field.

The combination of high-dimensional mobile sensing features and the flexibility of machine learning techniques allows for a data-driven, scalable, and personalized approach compared with traditional statistical methods [58-62]. Moreover, mobile sensing via smartphones or wearables offers a ubiquitous, continuous, and nonintrusive means of data collection, making it a powerful tool for capturing momentary experiences more effectively than traditional survey-based approaches [63-66]. Importantly, our goal is not to replace human expertise, but to augment it using technology for early detection and intervention at scale [67-69].

Passive detection of PUT presents unique challenges. Such events are often sporadic, vary greatly in form [70,71], and are perceived subjectively across individuals [72-74], leading to significant variability in experiences and reporting. This makes it difficult for longitudinal studies like ours to collect sufficient samples of day-to-day self-reported incidents, with adequate variance in the ground truth. Nonetheless, traditional instruments for measuring PUT, such as self-reports, have well-documented limitations [28], including biases [75,76] and limited ability to account for confounding factors such as physical or mental stressors. These tools also rely on repeated measures [77], which can lead to participant fatigue and reduced compliance over time. Importantly, self-reporting is inherently episodic, making it less suited for continuous, population-level screening across a campus setting. In contrast, passive sensing offers the ability to continuously and unobtrusively monitor behavioral and physiological signals over time, rendering it a promising tool to complement traditional methods in detecting and understanding these complex psychosocial phenomena.

Recent advancements in machine learning–based rare event detection (RED) have shown promising results across a range of domains, including health care and mobile sensing [78,79]. While traditional ensemble methods such as random forests and gradient boosting have been used [80-82], deep learning architectures such as autoencoders (AEs) and long short-term memory networks (LSTMs) are gaining momentum [83-88]. This is particularly relevant in multivariate time series settings [88,89], where smartphones and wearable devices continuously generate multiple streams of time-stamped data (eg, location, activity, and phone use) that capture complex behavioral patterns over time [90-93]. Despite these advancements, RED remains a challenging task [78,79], especially given the infrequent nature of the events. This often leads to reduced quantitative performance, particularly in metrics such as precision, recall, and *F*_1_-score, as studies across various domains frequently report only modest improvements over baseline methods [79]. For instance, Coley et al [94] reported a precision of 0.09, a recall of 0.53, and an *F*_1_-score of 0.16 using a random forest model for suicide risk detection on a health care dataset with a rarity of 0.2%. Closer to our context, Pillai et al [95] proposed a multitask learning framework in the Tesserae study [92] that combined an unsupervised AE with an auxiliary sequence prediction task to detect rare life events (<2%) using mobile sensing data. This approach improved performance compared with several baselines, achieving an *F*_1_-score of 0.29. Such approaches have not yet been explored for detecting PUT experiences in social contexts like college environments. This gap presents an important opportunity to investigate the potential of mobile sensing-based RED methods in screening behavioral anomalies that may signal experiences of PUT.

Building on prior work [57,95], we developed and evaluated machine learning models leveraging mobile sensing data collected in 2018 as part of a multiyear study of undergraduate students [93,96]. We focused on two modeling approaches: (1) user-independent classification models, trained to identify behavioral patterns that are indicative of PUT across individuals; and (2) user-dependent anomaly detection models, personalized to detect short-term deviations in individual behavior that may signal responses to such experiences. Our objective is to assess the feasibility of using exclusively mobile sensing data, collected up until the time students wake the next morning, to detect experiences of PUT from the previous day at both the population and individual levels. We evaluated model performance against naive baselines using metrics commonly used in RED [97], including area under the receiver operating characteristic curve (AUC-ROC), area under the precision-recall curve (AUC-PR), precision, recall, and *F*_1_-score.

## Methods

### Ethical Considerations

This work was approved by the University of Washington Institutional Review Board and was assigned the ID Study00003324. All participants in the study provided their informed consent in person. All data collected in the study were kept separate from participants’ personal identifiers, to provide anonymity and protect privacy. Participants could receive compensation up to US $245/quarter in gift cards, depending on the completeness of their data collection, both passively sensed data and EMAs.

### Data Collection

To be eligible for the study, participants were required to be over 18 years old, enrolled as first-year full-time undergraduates, and own an Android or iOS smartphone with an active data plan. The data collection period spanned 2 academic terms (January to June 2018), lasting approximately 20 weeks. At the beginning of the study, participants installed a smartphone app built using the AWARE framework [98] and wore a Fitbit tracker continuously throughout the study period, enabling passive data collection. This setup captured a wide range of mobile contextual information, including location, activity recognition, battery status, phone calls, screen use, and Bluetooth and Wi-Fi scans, while the Fitbit tracker provided steps and sleep data.

During the study, participants received regular EMA surveys [99] to report PUT experiences (Multimedia Appendix 1 contains survey questions). They completed EMAs twice weekly (on Sundays and Wednesdays) over an 8-week period, reporting on events from the previous day, and completed daily evening EMAs for an additional 2 weeks each academic term, reflecting on the same day's experiences. To identify which days participants experienced PUT, each EMA asked whether they had experienced unfair treatment on the reported day (today or yesterday).

### Feature Extraction

In this study, we computed behavioral features from 7 smartphone data streams (activity recognition, battery, Bluetooth, call, location, screen, and Wi-Fi) and 2 wearable data streams (sleep and steps). We extended a behavioral feature extraction library [100] and followed a similar approach to prior work [90,101] by aggregating mobile sensing data into statistical summaries across various epochs of the day, including night (12 AM to 6 AM), morning (6 AM to 12 PM), afternoon (12 PM to 6 PM), and evening (6 PM to 12 AM), as well as the entire day (12 AM to 12 AM). This approach allowed us to characterize human behavior patterns within the day, providing a structured representation of daily behavior. Additionally, aligning our feature extraction process with prior work ensured methodological consistency, enabling direct comparisons and potential cross-study generalization. Notably, sleep features were computed only on a daily basis, as most student participants typically experienced a single major sleep episode each night. Some features were stream-specific, such as location variance derived solely from GPS data or the frequency of screen unlocks from screen events. Others involved multiple streams, for example, estimating indoor mobility duration by fusing location and activity recognition data. We describe the extraction of each set of behavioral features in Multimedia Appendix 2. In addition to these features, we computed the number of data samples collected for each data stream as an additional feature. This allowed us to assess data compliance and gain insights into event-based data streams such as calls, screen use, activity, and sleep, where the number of records reflects the frequency of these events.

### Data Availability Analysis

Out of the 201 study participants, 110 individuals reported experiencing PUT at least once during the study period. To estimate how frequently PUT was reported overall, we calculated the proportion of positive EMA responses (indicating PUT) for each participant (ie, the number of positive responses divided by the total number of EMA responses they submitted). We then averaged these individual-level proportions across all participants, resulting in a mean reporting rate of 4.66% (95% CI 3.13% to 6.19%). The distribution of the EMA survey responses, with a total of 9629 submissions, included 413 (4.3%) positive responses and 9216 (95.7%) negative responses.

We observed a significant level of missing values in the behavioral features. Issues related to data collection, such as poor study compliance, phone battery depletion, or app crashes, directly contributed to the lack of raw sensing data. Additionally, event-based data streams, such as call logs, only record data when specific events occur (eg, when a call is made), making it challenging to determine whether the absence of data was due to the absence of such events (eg, no calls made) or due to issues in data collection. Insufficient volume of raw sensing data per time period can also result in missing feature values. This is especially relevant for statistical features that require a reasonable number of samples for aggregated calculations. Similarly, features like Bluetooth and location often rely on a sufficient number of raw data records for effective data clustering. Last, the limited diversity of data streams per sample can affect the computation of fused features, as they depend on nonmissing values from multiple data streams. For instance, both location and activity recognition data are required for extracting features such as study duration and indoor mobility.

In Table 1, we report the availability of each data stream in the raw dataset, which serves as the foundation for feature calculation. We computed data availability as the percentage of daily samples where each data stream was available, relative to the total samples across all participants (total days × total participants). A higher percentage indicates broader availability.

**Table 1:** Availability of each data stream in the raw dataset.

Data stream	Availability (%)
Activity	49.93
Battery	43.61
Bluetooth	48.65
Calls	35.05
Locations	51.08
Screen	51.85
Wi-Fi	52.44
Sleep	65.17
Steps	70.52

### Modeling

**Figure 1:**
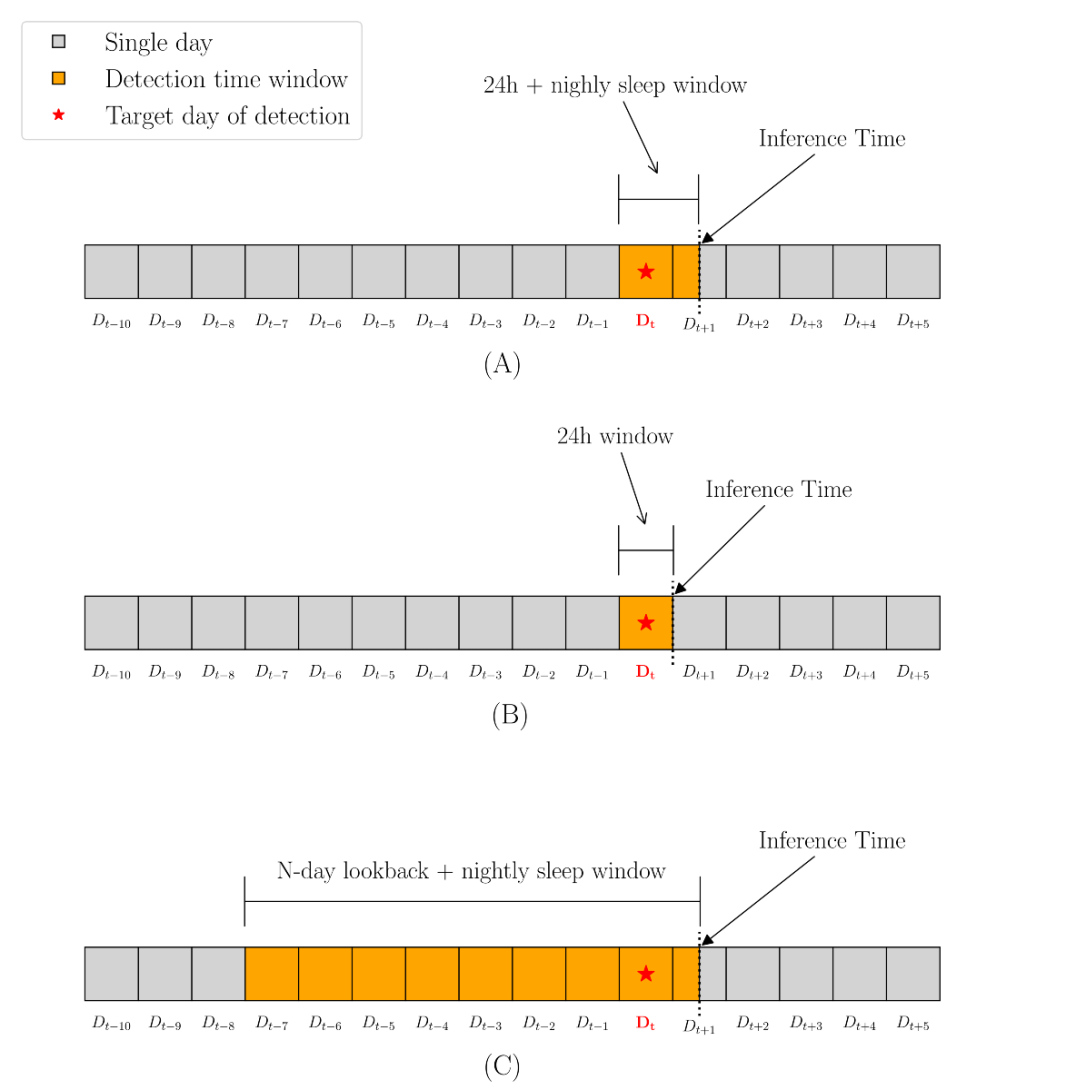
Illustration of detection time windows and inference times for three model types: (A) user-independent classification model, (B) intraday long short-term memory-autoencoder user-dependent model, and (C) multiday long short-term memory-autoencoder user-dependent model.

In this study, we focus on 2 modeling approaches (user-independent and -dependent) to retrospectively detect PUT using daily inference windows (Figure 1), implemented through 3 specific model types. The user-independent classification models leverage labeled data from training participants to detect PUT in new or unseen participants. In contrast, the user-dependent anomaly detection models learn from participants’ historical data to differentiate between normal and rare patterns in their own behavior. These approaches are driven by practical considerations. First, the scarcity of labeled data makes it difficult to build a model that generalizes well to unseen users [61,93,96,102-105], though identifying broad patterns and key features remains essential in imbalanced settings [106]. Second, individuals' behavioral patterns before and after experiencing PUT can vary greatly [21,107-110] and are often moderated by various factors [111-115], highlighting the need for personalized training strategies. Third, given the rarity of target events, modeling them as anomalies is a commonly adopted approach in a wide variety of studies [78,79]. Furthermore, we believe these dual modeling approaches enhance the robustness of our models across diverse real-world deployment scenarios.

### User-Independent Modeling

We selected light gradient boosting machine (LightGBM) [116] as our primary algorithm for user-independent modeling, for its ability to handle high-dimensional mobile sensing features, capture nonlinearity, mitigate overfitting through its ensemble mechanism, and natively manage missing data. LightGBM is a gradient boosting framework that builds decision trees efficiently, using a histogram-based approach to speed up training while maintaining high accuracy. Its built-in feature importance calculation enhances model interpretability and makes it an ideal choice for gaining population-wide insights in imbalanced settings. We benchmarked LightGBM with 4 classic machine learning algorithms: k-nearest neighbors (KNN), logistic regression, support vector machine (SVM), and random forest. All models were implemented using the scikit-learn Python library [117], with binary cross-entropy loss as the objective function. For comparison, we also implemented 3 baseline classifiers. The first uses only static demographic information (Multimedia Appendix 3) without incorporating any behavioral data. The other two are naive baselines, which make random predictions without considering input features: (1) a uniform classifier that assigns labels randomly with equal probability, ignoring class distribution; and (2) a stratified classifier that assigns labels randomly while preserving the class distribution observed in the data.

Prior to training, we applied a filtering step that required each sample to contain at least 7 available data streams, resulting in a final dataset of 4720 person-day records. Of these, 167 (3.5%) were labeled positive and 4553 (96.5%) as negative. Participants contributed an average of 24 days of usable data (SD 9.6). LightGBM natively handled missing values, while for the classic models, we applied median imputation via scikit-learn’s SimpleImputer. To address class imbalance, we experimented with class weight adjustments as well as 2 widely used oversampling approaches: the synthetic minority oversampling technique and SVM-based synthetic minority oversampling technique [118,119].

We chose the best-performing population-level model for subsequent feature analysis and selection, which was then used to build user-dependent models. As illustrated in Figure 1A, all models were trained on features extracted from a full 24-hour period on the target day, combined with sleep features from the nightly sleep window ending the following morning, after which inference was performed.

### User-Dependent Modeling

**Figure 2:**
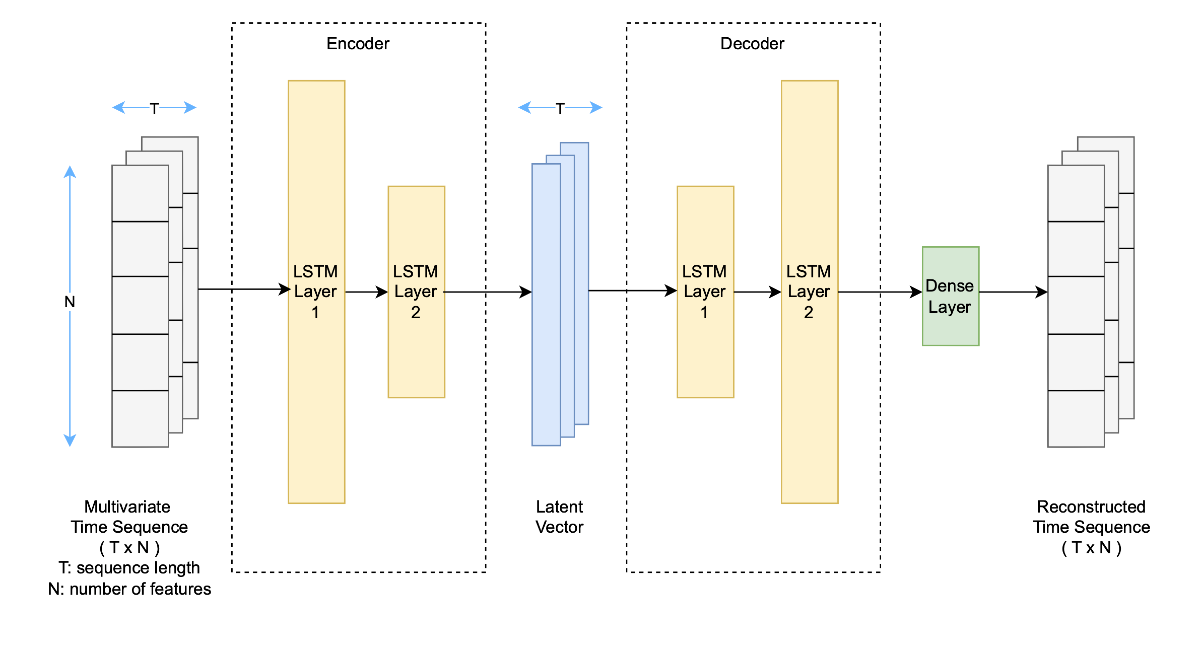
LSTM autoencoder architecture. LSTM: long short-term memory.

For user-dependent modeling, we focused on anomaly detection with LSTM-AEs. LSTM-AEs are a type of neural network designed to learn temporal patterns in sequential data by encoding and reconstructing time-series inputs, making them effective for detecting deviations from typical behavioral patterns. Since human behavior typically follows daily routines and longer-term cycles (eg, weekly), we believe this approach helps detect anomalies in individual trajectories that may signal PUT. Our LSTM-AE architecture (Figure 2) consists of 2 stacked LSTM layers (encoder) that compress input sequences into a fixed-size representation, which is then replicated and decoded by another 2 LSTM layers (decoder). A final dense layer reconstructs the original sequence. The model learns normal behavioral patterns by minimizing reconstruction loss (mean squared error). We implemented the model architecture using Keras and TensorFlow libraries. We explored both intraday (1-day lookback) and multiday (N-day lookback) input constructions to assess whether behavioral changes associated with PUT are better detected through short-term or longer-term temporal context. The intraday model focuses on capturing short-term daily patterns by dividing each 24-hour period into 4 fixed 6-hour epochs: night (12 AM to 6 AM), morning (6 AM to 12 PM), afternoon (12 PM to 6 PM), and evening (6 PM to 12 AM), resulting in sequences with 4 aggregated data points. In contrast, the multiday model captures longer-term trends using an N-day lookback window, where each sequence includes N days of daily features, ending with the target day. For both intraday and multiday models, we selected features based on the top behavioral features identified by the best-performing user-independent model. For the intraday model, inference occurs daily at the end of the day (Figure 1B); whereas for the multiday model, inference is performed the following morning after sleep-related features are available (Figure 1C). During inference, anomaly scores for intraday sequences were derived from the reconstruction loss of the entire sequence, whereas for multiday sequences, only the loss of the target (last) day was used. Anomalies were then defined using a threshold set at the 75th percentile of training reconstruction errors, providing a conservative and data-driven decision rule.

The models were trained exclusively on negative samples, with all positive samples reserved for testing, which is a common practice in RED [120-122]. Negative samples were split chronologically (90:10) into training and test sets. The training and test sample sizes are shown in Table 2. To prevent information leakage, multiday training samples overlapping with positive test samples were excluded. To ensure data quality, input sequences with more than 80% missing data were removed, and for multiday models, sequences with more than 50% missingness on the target day were also excluded. Reconstruction loss was computed only for observed (non-NaN) positions to minimize bias.

**Table 2:** Training and test sample sizes for user-dependent modeling with long short-term memory autoencoders.

Model		Train (Neg)	Test			Per user	
			Pos	Neg	Train, mean (SD)		Test, mean (SD)
**Intraday models**							
	50 features	6595	311	833	33 (12)		6 (3)
	100 features	6558	309	827	33 (12)		6 (3)
	150 features	4659	199	618	23 (8)		4 (3)
**Multiday models**							
	50 features, 3-day window	4428	191	598	22 (9)		4 (3)
	50 features, 5-day window	4341	191	598	22 (9)		4 (3)
	50 features, 7-day window	4299	190	596	21 (9)		4 (3)
	50 features, 9-day window	4248	189	596	21 (9)		4 (3)

Additionally, for comparison, we implemented two naive baseline models: (1) a uniform classifier that assigns labels randomly with equal probability, ignoring class distribution; and (2) a stratified classifier that assigns labels randomly while preserving the class distribution observed in the data.

### Model Evaluation

For the user-independent model, we implemented nested stratified group K-fold cross-validation to address the dataset’s limited size and class imbalance. The outer loop (*K*=5) split the data into training and test sets, ensuring class balance and participant-level separation. The inner loop (*K*=4) further splits the training data for feature selection and hyperparameter tuning, following the same stratified, participant-dependent structure. We evaluated performance using threshold-independent metrics (AUC-PR and AUC-ROC) and threshold-dependent metrics (precision, recall, and *F*_1_-score), reporting the mean and standard deviation across 25 independent performance estimates obtained from 5 repeats of 5-fold cross-validation with different random seeds. Threshold-dependent metrics are sensitive to both the chosen cutoff and outcome prevalence. To address this, we set the classification threshold to the empirical positive class probability from the training data, providing a consistent and data-driven operating point. Threshold-independent metrics complement this choice by characterizing performance across all thresholds, with AUC-PR being particularly informative under class imbalance [123,124]. Together, these metrics allow both robust model comparison and an understanding of the practical trade-offs between capturing rare events and limiting false alarms.

For the user-dependent anomaly detection model, we likewise evaluated performance using threshold-independent metrics (AUC-PR and AUC-ROC) and threshold-dependent metrics (precision, recall, and *F*_1_-score), reporting the mean and standard deviation across 10 randomized runs with different model initializations. During inference, the anomaly threshold was conservatively set at the 75th percentile of training reconstruction errors, as the limited sample size precluded further fine-tuning. Threshold-independent metrics were computed from anomaly scores across test samples, while threshold-dependent metrics reflected performance at the selected threshold.

## Results

### Participants’ Characteristics

The study focused on 201 full-time undergraduate students from the cohort enrolled in 2018 at the University of Washington. The mean age of the sample was 18.4 (SD 0.56) years. Female students comprised 64.7% (130/201 students) of the sample. Academically, students were drawn from a variety of departments, with approximately half majoring in engineering. To ensure a diverse sample, recruitment strategies included the oversampling of students from underrepresented backgrounds, specifically those with disabilities, first-generation students, and gender minorities.

### User-Independent Modeling Results

Table 3 reports the performance metrics of all user-independent classification models alongside the baseline classifiers. To assess whether benchmarked models significantly outperformed the baselines, we conducted pairwise comparisons using the Wilcoxon signed-rank test (paired, 1-sided). Table 4 presents the *P* values from these comparisons, adjusted using the Benjamini-Hochberg false discovery rate procedure. The classification models showed mixed performance, with AUC-ROC ranging from 0.546 to 0.640, AUC-PR from 0.047 to 0.093, and *F*_1_-scores from 0.070 to 0.121. Compared with the baselines, some benchmarked models showed modest improvements. Both Random Forest and LightGBM achieved higher AUC-ROC and AUC-PR scores compared with KNN, Logistic Regression, SVM, and the baseline classifiers. Among all models, LightGBM achieved the highest *F*_1_-score. The observed variability across the cross-validation folds highlights the challenge of between-individual generalizability.

**Table 3:** Performance of user-independent classification models compared with baseline classifiers. Metrics are reported as mean (SD) across 25 estimates from repeated 5-fold cross-validation (5 repeats, different random seeds).

Model			AUC-ROC^a^, mean (SD)		AUC-PR^b^, mean (SD)	Precision, mean (SD)		Recall, mean (SD)	*F*_1_-score, mean (SD)
**Baseline models**									
	Uniform	0.500 (0.000)		0.036 (0.018)		0.034 (0.018)	0.492 (0.121)		0.063 (0.031)
	Stratified	0.501 (0.022)		0.037 (0.018)		0.046 (0.048)	0.041 (0.040)		0.039 (0.036)
	Demographic	0.523 (0.126)		0.057 (0.043)		0.051 (0.044)	0.267 (0.143)		0.083 (0.065)
**User-independent models**									
	KNN^c^	0.561 (0.062)		0.049 (0.025)		0.037 (0.020)	0.836 (0.074)		0.070 (0.036)
	Logistic Regression	0.546 (0.082)		0.047 (0.026)		0.041 (0.023)	0.579 (0.145)		0.075 (0.040)
	SVM^d^	0.567 (0.095)		0.065 (0.045)		0.053 (0.039)	0.268 (0.139)		0.089 (0.056)
	Random Forest	0.634 (0.086)		0.093 (0.094)		0.045 (0.022)	0.709 (0.143)		0.084 (0.038)
	LightGBM^e^	0.640 (0.065)		0.077 (0.043)		0.083 (0.050)	0.275 (0.118)		0.121 (0.064)

^a^AUC-ROC: area under the receiver operating characteristic curve.

^b^AUC-PR: area under the precision-recall curve.

^c^KNN: k-nearest neighbors.

^d^SVM: support vector machine.

^e^LightGBM: light gradient boosting machine.

**Table 4:** Results of Wilcoxon signed-rank tests comparing user-independent models with baseline models. Reported *P* values were obtained from paired, one-sided Wilcoxon signed-rank tests and adjusted for multiple comparisons using the Benjamini-Hochberg false discovery rate (FDR) correction.

Baseline models		*P* values				
		AUC-ROC^a^	AUC-PR^b^	Precision	Recall	*F*_1_-score
**KNN^c^**						
	Uniform	<.001	<.001	.03	<.001	.004
	Stratified	<.001	<.001	.72	<.001	.002
	Demographic	.16	.88	.97	<.001	.79
**Logistic regression**						
	Uniform	.007	<.001	.004	.006	.003
	Stratified	.007	<.001	.57	<.001	<.001
	Demographic	.33	.91	.96	<.001	.65
**SVM^d^**						
	Uniform	.002	<.001	.002	>.99	.004
	Stratified	.003	<.001	.16	<.001	<.001
	Demographic	.16	.34	.56	.67	.54
**Random forest**						
	Uniform	<.001	<.001	<.001	<.001	<.001
	Stratified	<.001	<.001	.41	<.001	<.001
	Demographic	.002	.03	.72	<.001	.23
**LightGBM^e^**						
	Uniform	<.001	<.001	<.001	>.99	<.001
	Stratified	<.001	<.001	.002	<.001	<.001
	Demographic	<.001	.03	.003	.47	.013

^a^AUC-ROC: area under the receiver operating characteristic curve.

^b^AUC-PR: area under the precision-recall curve.

^c^KNN: k-nearest neighbors.

^d^SVM: support vector machine.

^e^LightGBM: Light Gradient Boosting Machine.

The pairwise comparisons indicated that both LightGBM and Random Forest outperformed all 3 baselines in terms of AUC-ROC and AUC-PR. LightGBM improved AUC-ROC by 22.4% (0.640 vs 0.523, *P*<.001) and Random Forest improved AUC-PR by 63.2% (0.093 vs 0.057, *P*=.03), both relative to the demographic baseline. While KNN, Logistic Regression, and Random Forest achieved significantly higher recall than the baselines, their precision values were significantly lower. LightGBM showed significant improvements in both precision and *F*_1_-score compared with all 3 baselines, albeit at the cost of lower recall. Other benchmarked models showed smaller or nonsignificant differences, particularly compared with the demographic baseline. Overall, these findings suggest that tree-based models can improve the detection of PUT events, which we explore further in the Discussion.

### User-Dependent Modeling Results

#### Intraday Models

**Figure 3:**
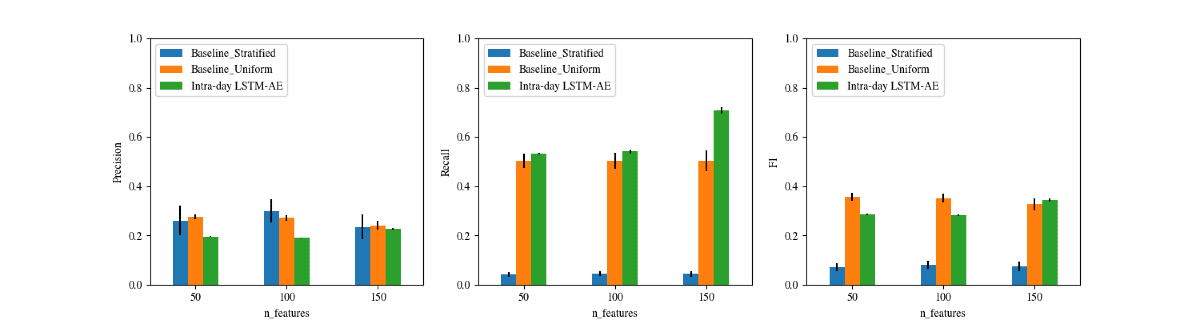
Performance of the intraday anomaly detection model using varying numbers of input features, compared against naive baseline models. LSTM-AE: long short-term memory-autoencoder.

Figure 3 plots the performance of the intraday LSTM-AE model as the number of input features increases, compared with 2 naive baselines. Models trained with 50 and 100 features did not yield improvements over the baselines except for recall, while the model trained with 150 features showed improvements in both recall and *F*_1_-score. In general, models with more input features achieved higher recall.

#### Multiday Models

**Figure 4:**
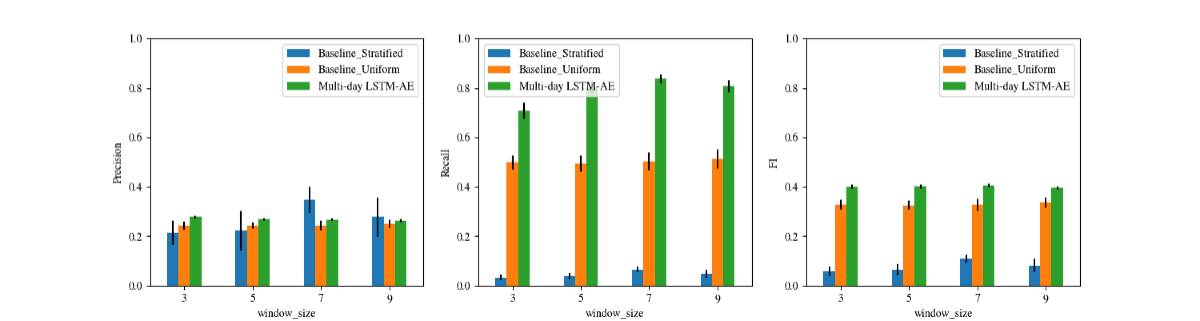
Performance of the multiday anomaly detection model across different window sizes, compared against naive baseline models. LSTM-AE: long short-term memory-autoencoder.

Figure 4 plots the performance of the multiday LSTM-AE model trained on input sequences with 50 features across varying window sizes (3, 5, 7, and 9 days), compared with 2 naive baselines. All models consistently outperformed both the stratified baseline and the uniform baseline with respect to recall and *F*_1_-score (*P*<.001 for both). The model with a 7-day window achieved the highest recall (0.836), while the 3-day model achieved the highest precision (0.279). All multiday LSTM-AE models had comparable *F*_1_-scores (0.397-0.405). The 9-day window model showed a slight decline relative to the 7-day model in both recall (0.807 vs 0.836) and precision (0.397 vs 0.405), suggesting that extending the temporal window beyond 7 days yields diminishing returns.

Table 5 summarizes the performance of the best-performing intraday and multiday models, alongside their respective naive baselines for reference. To assess whether benchmarked models significantly outperformed the baselines, we conducted pairwise comparisons using the Wilcoxon signed-rank test (paired, one-sided). Table 6 presents the *P* values from these comparisons, adjusted using the Benjamini-Hochberg false discovery rate procedure. As shown in Tables 5 and 6, the intraday model (150 features) did not show significant improvements over baselines except for recall (*P*<.001) and *F*_1_-score (*P*=.012 vs uniform; *P*<.001 vs stratified). In contrast, the multiday model (50 features, 7-day window) significantly outperformed both baselines on nearly all evaluation metrics, except for precision against the stratified baseline. It achieved significantly higher recall (0.830 vs 0.479 for the uniform baseline, +73.3%, *P*<.001) and *F*_1_-score (0.391 vs 0.313 for the uniform baseline, +24.9%, *P*<.001) with comparable precision (0.256), while improving AUC-PR by 45.9% (0.353 vs 0.242, *P*=.002) and AUC-ROC by 21.6% (0.605 vs 0.500, *P*=.002) relative to the baselines.

**Table 5:** Performance of user-dependent models compared with baseline classifiers. Metrics are reported as mean (SD) across 10 randomized runs with different model initializations.

Model			AUC-ROC^a^, mean (SD)		AUC-PR^b^, mean (SD)		Precision, mean (SD)		Recall, mean (SD)	*F*_1_-score, mean (SD)
**Intraday (150 features)**										
	Baseline (“uniform”)	0.500 (0.000)		0.244 (0.000)		0.243 (0.013)		0.495 (0.030)		0.326 (0.017)
	Baseline (“stratified”)	0.500 (0.000)		0.244 (0.000)		0.263 (0.069)		0.046 (0.016)		0.078 (0.027)
	LSTM-AE^c^	0.447 (0.004)		0.240 (0.002)		0.227 (0.003)		0.703 (0.010)		0.343 (0.005)
**Multiday (50 features, 7-day window)**										
	Baseline (“uniform”)	0.500 (0.000)		0.242 (0.000)		0.233 (0.015)		0.479 (0.034)		0.313 (0.020)
	Baseline (“stratified”)	0.500 (0.000)		0.242 (0.000)		0.250 (0.079)		0.044 (0.014)		0.074 (0.024)
	LSTM-AE	0.608 (0.008)		0.353 (0.008)		0.256 (0.005)		0.830 (0.027)		0.391 (0.007)

^a^AUC-ROC: area under the receiver operating characteristic curve.

^b^AUC-PR: area under the precision-recall curve.

^c^LSTM-AE: long short-term memory-autoencoder.

**Table 6:** Results of the Wilcoxon signed-rank tests comparing user-dependent models with baseline models. Reported *P* values were obtained from paired, one-sided Wilcoxon signed-rank tests and adjusted for multiple comparisons using the Benjamini-Hochberg false discovery rate (FDR) correction.

Baseline			*P* values								
			AUC-ROC^a^		AUC-PR^b^		Precision		Recall		*F*_1_-score
**Intraday LSTM-AE^c^ (150 features)**											
	Uniform	>.99		>.99		>.99		<.001		.012	
	Stratified	>.99		>.99		>.99		<.001		<.001	
**Multiday LSTM-AE (50 features, 7-day window)**											
	Uniform	.002		.002		<.001		<.001		<.001	
	Stratified	.002		.002		.99		<.001		<.001	

^a^AUC-ROC: area under the receiver operating characteristic curve.

^b^AUC-PR: area under the precision-recall curve.

^c^LSTM-AE: long short-term memory-autoencoder.

### Feature Importance Analysis

Analyzing feature importance in user-independent classification models is essential for identifying key behavioral features associated with reported PUT at the population level. In this study, we examined feature importance scores from the best-performing LightGBM model computed using information gain and averaged across all cross-validation folds. Higher scores indicate greater influence on the model’s decision. However, feature importance in tree-based models can be sensitive to correlated features [125] or sampling imbalance [126] and should be interpreted with caution. Future work could incorporate Shapley additive explanations [127] values to provide more robust insights into feature contributions and interactions in model decisions.

Table 7 summarizes the top 30 daily behavioral features ranked by their average importance scores. To better understand their associations with the outcome, we analyzed value distributions across positive and negative daily samples and computed standardized mean differences (SMDs) as effect sizes. Larger SMDs indicate stronger class separation. All top-ranked features had |SMD| values below 0.5, with 17 falling in the small-to-moderate range (|SMD| between 0.2 and 0.5) and 13 in the minimal separation range (|SMD| less than 0.2). This indicates that while these features may not independently distinguish between the 2 outcomes, they likely contribute to the model's decision through complex interactions or nonlinear associations.

Interestingly, the top 10 features are evenly distributed across a variety of sensing modalities, including 2 from steps, one fused feature derived from both activity and location, and one feature each from the remaining 6 data streams: location, activity, screen, Bluetooth, Wi-Fi, and call. This balanced distribution suggests that diverse behavioral signals, rather than any single dominant source, collectively contribute to the model’s detection ability. Across the top 30 features, the most frequently represented time epochs were the all-day window (n=9) and the evening period (n=7), indicating that both cumulative and evening-specific behavioral patterns are particularly informative for detection. As summarized in Textbox 1, these selected features touch upon key aspects of students’ daily lives, reflecting mobility, activity, phone use, social interactions, and sleep patterns.

**Table 7:** Top 30 daily behavioral features identified by the best-performing light gradient boosting machine model.

Feature	Data stream	Epoch	Positive, mean (SD)	Negative, mean (SD)	Importance score	Effect size (SMD^a^)
Total time spent off-campus (minutes)	Location	All day	729.5 (505.8)	535.3 (435.3)	892.1	0.44
Total step count	Steps	Evening	4140.6 (2878.8)	3106.0 (2726.1)	628.5	0.38
Activity sample count	Activity	Night	171.7 (102.4)	139.6 (96.2)	595.8	0.33
Total screen time (minutes)	Screen	Afternoon	116.7 (70.1)	90.1 (64.9)	447.9	0.41
Bluetooth sample count	Bluetooth	Morning	25.2 (76.1)	45.9 (106.0)	415.3	–0.20
Number of unique Wi-Fi access points	Wi-Fi	All day	17.8 (16.9)	17.7 (24.0)	380.1	0.01
Number of missed calls	Call	Evening	1.2 (1.9)	0.8 (1.6)	290.2	0.25
Indoor mobility duration (minutes)	Activity/location	All day	48.3 (64.0)	65.2 (72.1)	289.2	–0.24
Sleep sample count	Sleep	All day	456.6 (128.9)	469.4 (112.0)	273.5	–0.11
Average duration of sedentary bouts (minutes)	Steps	Evening	37.8 (74.8)	37.2 (69.9)	257.2	0.01
Total number of active bouts	Steps	All day	56.9 (29.4)	52.3 (21.1)	192.2	0.21
Longest stay duration at study places (minutes)	Location	All day	50.6 (98.6)	45.4 (79.6)	175.5	0.06
Average stay duration off-campus (minutes)	Location	All day	137.6 (207.9)	91.1 (160.8)	175.4	0.29
Shortest phone interaction bout (minutes)	Screen	All day	10.5 (105.4)	3.5 (46.4)	173.2	0.14
First unlock time (seconds since midnight)	Screen	Night	1244.6 (2611.8)	1436.3 (3400.3)	159.2	–0.06
Average stay duration in green spaces (minutes)	Location	All day	27.4 (57.1)	26.1 (68.1)	146.4	0.02
Total sedentary time (minutes)	Steps	Evening	289.1 (48.9)	306.0 (40.4)	146.2	–0.42
Percentage of time spent off-campus	Location	All day	0.6 (0.4)	0.4 (0.3)	141.9	0.32
Shortest phone interaction bout (minutes)	Screen	Night	14.1 (44.2)	6.9 (34.3)	140.7	0.21
Activity sample count	Activity	Morning	203.2 (94.6)	211.0 (138.1)	135.1	–0.06
Sedentary bout duration variation (minutes)	Steps	Evening	28.5 (25.6)	29.8 (24.2)	132.8	–0.05
Circadian movement	Location	Night	2.3 (0.0)	2.3 (0.0)	132.7	0.02
Duration of physical activities (minutes)	Activity	Evening	84.8 (64.3)	64.9 (53.9)	131.9	0.37
Number of unique Wi-Fi access points	Wi-Fi	Afternoon	8.8 (8.6)	9.7 (38.8)	130.6	–0.03
Total time spent off-campus (minutes)	Location	Morning	171.1 (146.6)	117.8 (125.5)	130.0	0.42
Average steps per active bout	Steps	Evening	209.7 (137.0)	169.8 (141.0)	128.8	0.28
Last active bout end time (seconds since midnight)	Steps	Morning	40,457.5 (4216.2)	41,539.9 (2925.4)	127.9	–0.36
Variation in time spent in green spaces (minutes)	Location	Morning	3.2 (11.3)	1.3 (7.6)	120.9	0.23
Start time of Nightly sleep (seconds since midnight)	Sleep	All day	34,585.9 (7774.7)	33,569.8 (8917.1)	120.5	0.11
Shortest phone interaction bout (minutes)	Screen	Afternoon	1.6 (18.9)	2.3 (19.8)	118.8	–0.03

^a^SMD: standardized mean difference.

**Examples of selected sensing features and their relation to students’ daily behaviors.**

- Campus-map features: Features such as time spent off-campus and indoor mobility show that students who experience perceived unfair treatment (PUT) tend to spend more time away from campus and exhibit reduced movement within campus buildings.
- Physical activity features: Features such as step count and activity duration show that these students tend to be more active during the evening and night hours.
- Screen use features: Longer afternoon screen time, longer minimum interaction durations, and earlier phone unlocks at night among students who report experiences of PUT. These features provide insights into phone use patterns and digital engagement.
- Bluetooth features: Lower sample counts in the morning for students who experience PUT. These features may serve as indicators of social exposure and proximity to others.
- Call features: A higher number of missed calls during evening hours among students reporting PUT. These features may reflect phone availability and social responsiveness.
- Sleep features: Shorter sleep durations and later sleep onset times on days marked by PUT.

## Discussion

### Principal Findings

In this study, we reported results from both classification and anomaly detection machine learning models evaluated under user-independent and user-dependent settings for detecting PUT among college students. A novel aspect of our work is the exclusive use of passively collected mobile sensing data for training and inference, offering a nonintrusive and low-burden alternative to traditional self-reports. As shown in Figure 1, our models were able to detect past events within a day of occurrence (including the subsequent nightly sleep window), indicating that mobile sensing may offer timely detection of PUT experiences and potential practical applications in future interventions. The best-performing classification model (LightGBM) significantly outperformed all 3 baseline classifiers, including the demographic baseline, in AUC-ROC, AUC-PR, precision, and *F*_1_-score. The top-performing anomaly detection model, which incorporated a 7-day temporal context and 50 features, significantly outperformed the baselines across most metrics, achieving notably higher recall while maintaining comparable precision. These results suggest that mobile sensing features may serve as behavioral indicators for detecting experiences of PUT. While not intended to replace traditional assessments, mobile sensing could potentially complement existing methods, especially for large-scale or continuous monitoring.

Among the user-independent classification models, ensemble tree-based classifiers such as random forest and LightGBM consistently outperformed traditional machine learning algorithms like logistic regression and SVM, achieving higher overall performance. This result is consistent with previous work from various disciplines, highlighting the robustness of ensemble methods for handling imbalanced datasets [128-132] and the advantage of nonlinear models in capturing the complex behavioral patterns typically observed in mobile sensing data [90,93,133,134]. However, the overall limited performance across all models, along with the observed high variability across different user-independent data splits (eg, higher standard deviations in performance metrics), underscores the challenges of between-individual generalizability posed by the infrequent and subjective nature of the detection task. While user-independent models remain valuable for identifying globally informative features, future work should focus on personalized or semipersonalized modeling approaches that can better accommodate individual variability. Balancing scalability with personalization, for instance, by deploying personalized models for individuals identified as high-risk by a global model, may guide a more effective and targeted detection framework.

Our preliminary results suggest that daily behavioral signals alone, whether modeled in a user-independent setting (eg, classification models using daily features) or a user-dependent setting (eg, the intraday LSTM-AE model), were insufficient to reliably capture behavioral shifts associated with PUT. In contrast, models that incorporated multiday temporal windows demonstrated notable performance improvements. For example, all multiday anomaly detection models achieved statistically significant gains in recall and *F*_1_-score compared with the corresponding baselines. This suggests that a longer temporal context may help detect more gradual or subtle behavioral deviations that may not be evident within a single day. Among the multiday models, the 7-day model achieved the best overall performance, while longer windows (eg, 9-day) showed reduced effectiveness. This suggests that the 7-day window likely strikes a balance between capturing sufficient behavioral context and remaining short enough to detect localized anomalies. It may also align with natural weekly rhythms that help reveal meaningful patterns. Taken together, these findings highlight the potential value of modeling temporal dynamics across multiple days. We recommend that future work identify and apply an optimal window length that balances contextual richness with anomaly detectability to improve sensitivity without compromising precision.

In user-dependent anomaly detection, intraday models with 150 features outperformed those with 50 or 100 features, underscoring the value of a rich feature set for capturing behavioral anomalies. Feature importance analysis further indicated that performance gains were not driven by any single type of sensor data. Instead, a diverse set of behavioral indicators spanning multiple sensing modalities, including physical activity, phone use, mobility, and sleep, all contribute meaningfully to model performance. The absence of a dominant modality or feature suggests that effective detection likely relies on multimodal inputs and potentially their interactions to capture the complex, context-dependent nature of behavioral patterns rather than on isolated signals. These findings highlight the multifaceted nature of behavioral responses to PUT, though larger studies are needed to confirm these patterns. In the following section, we further contextualize these results by relating them to prior work.

### Connections to Existing Literature

Our location-based features reveal that on days with reported PUT, students spend more time off-campus, which may reflect disengagement or withdrawal from campus life. Indoor mobility within campus buildings is also lower (eg, fewer transitions between classrooms, libraries, or other study areas), potentially indicating reduced academic or social participation. Moreover, Bluetooth sample counts are lower on these days, revealing fewer nearby Bluetooth-enabled devices, which may imply decreased social exposure. These findings support prior research showing that discrimination can undermine students’ sense of belonging and social well-being, often resulting in increased feelings of isolation, social withdrawal, decreased academic and campus engagement, and even truancy [27,135-139]. Regarding physical activity, our features show that evening and nighttime activity tends to be higher on days with reported PUT, with greater step counts and longer durations of physical activity. These findings align with previous studies linking higher physical activity to perceived discrimination [109,140,141], possibly reflecting altered routines or coping behaviors. Our phone-use related features show longer afternoon screen time, earlier first unlocks, and longer shortest nightly phone interactions on days with reported PUT compared with days without. These patterns align with prior findings linking perceived discrimination to problematic phone use among students [142-144]. Finally, our sleep features show that students reporting PUT tend to have a later sleep onset and shorter sleep duration on the same day. This is consistent with previous research linking perceived discrimination to reduced sleep duration and poorer sleep quality [145-150].

While closely aligning with prior research, our study extends the literature by offering a unique short-term behavioral lens on the impact of PUT, complementing the longer-term patterns typically emphasized in the field. Many of the sensed patterns we identify likely reflect students’ immediate behavioral and physiological responses to PUT. These patterns are not only informative for detection but can also guide interventions in campus environments, enabling continuous monitoring and timely, targeted support. While our models cannot prevent the initial event, they could facilitate timely interventions to mitigate its adverse effects and promote student well-being. For instance, our models could trigger personalized microinterventions delivered via push notifications [151-154], such as encouraging on-campus engagement, prompting positive social interaction, suggesting phone-use breaks, offering early sleep reminders and sleep hygiene education. These data-driven strategies can be integrated with traditional approaches, including support groups, self-care practices, and professional mental health services.

### Cross-Domain Reflection: Rare Event Detection Challenges

We explored applying RED methodologies to the domain of detecting daily PUT. While we were able to connect some of our findings to existing literature on discrimination, most studies focused on retrospective self-reports, prevalence, and associated health outcomes. Relatively little attention has been given to detecting these events as they occur in daily life. To better situate our methodology and evaluate our model’s performance, we conducted a comparative review of established RED approaches across diverse domains, including health care, crowd behavior, and mechanical systems. As summarized in Multimedia Appendix 4, these studies [94,95,155-158] use a wide range of methods and demonstrate anomaly detection performance broadly comparable to ours.

Although our research is situated in a different domain, we find significant value in such cross-domain reflection. First, it allows us to assess whether our progress in capturing PUT experiences aligns with the advancements achieved in other novel contexts. Second, it helps validate our modeling approaches. Given the novelty of our application, we view these comparisons as reflective benchmarks rather than direct performance evaluations.

In our work, we encountered several challenges inherent to RED, including a highly skewed class distribution, difficulties in capturing correlations due to data sparsity, and similarities between rare and nonrare events. These challenges are consistent with those faced by other RED studies across various domains [79]. A further commonality is the inherent trade-off between recall and precision. High recall indicates a model’s ability to capture rare instances, but it is often accompanied by lower precision, suggesting that many predicted events may not be actual occurrences. This pattern is evident not only in our results but also across other domains. For instance, Pillai et al [95], in a context most similar to ours, achieved a recall of 0.21 and a precision of 0.47 using a dataset where rare events constituted approximately 1.9% of the data. In health care, however, models often deliberately favor recall over precision to ensure that critical cases are not missed. Inspired by this perspective, our study advocates for a recall-oriented approach that prioritizes identifying as many PUT experiences as possible, thereby maximizing opportunities to support students at risk.

A distinguishing aspect of our work is the relatively small dataset compared with those used in other studies, such as Coley et al [94] in suicide risk assessment. Despite this limitation, our models achieved recall and *F*_1_-scores that are comparable to, or in some cases exceed, those reported in similar domains, although direct comparisons are limited by dataset differences.

### Limitations

We recognize several limitations in this pilot study. First, responses to PUT were collected at the daily level in a binary format (yes or no), without capturing the exact timing of each incident. This limited our ability to precisely align intraday sensor data patterns with specific experiences. For this reason, we cannot rule out that the intraday model’s performance was affected by this limitation. Future work could benefit from more granular reporting to enable more accurate temporal analyses.

Second, our dataset was relatively small. While adequate for this exploratory pilot study, larger and more diverse datasets will be needed to validate and generalize these findings.

Third, this study focused exclusively on mobile sensing features. While this approach allowed us to isolate the predictive utility of passive sensing data, we acknowledge that the integration of additional domain knowledge, such as demographic and socioeconomic variables, or information about the type or reason for each incident, may improve model performance, interpretability, and fairness. Future research could reintroduce demographic and contextual information to account for heterogeneity across individuals and potentially enhance both predictive accuracy and fairness.

Finally, while our study EMAs were designed to directly ask about the target event and we aimed to detect PUT based on participants’ behavioral responses, we acknowledge that many of the identified behavioral patterns (eg, social withdrawal, changes in phone use, sleep, and physical activity) could result from other negative experiences or mental health conditions such as depression, representing potential confounding factors. In addition, we cannot rule out the potential moderating influence of other variables on the association between PUT and the sensed features. Fully disentangling PUT-related signals from overlapping influences remains an open challenge and an important direction for future research.

### Conclusions

This pilot study demonstrates the feasibility of using mobile sensing to screen for instances of PUT (4.3% or 413 of 9629 responses) among college students, providing a promising alternative to traditional self-report methods. Our machine learning models, leveraging diverse mobile sensing features and multiday temporal context, show strong potential for capturing short-term behavioral changes indicative of these infrequent experiences. We envision that future personalized and context-aware ML approaches, enhanced by larger datasets and deeper domain knowledge, will further improve detection accuracy, ultimately enabling timely interventions and support for at-risk students.

## Appendix

Multimedia Appendix 1 Ecological momentary assessment questions.

Multimedia Appendix 2 Behavioral feature extraction details.

Multimedia Appendix 3 Demographic variables used in baseline classifier.

Multimedia Appendix 4 Comparison of rare event detection studies.

## Abbreviations

AE: autoencoder
AUC-PR: area under the precision-recall curve
AUC-ROC: area under the receiver operating characteristic curve
EMA: ecological momentary assessment
KNN: k-nearest neighbors
LightGBM: light gradient boosting machine
LSTM: long short-term memory
PUT: perceived unfair treatment
RED: rare event detection
SMD: standardized mean difference
SVM: support vector machine

## Data Availability

The datasets generated or analyzed during this study are available in the GLOBEM repository [159].
