# Supplementary material for: Detecting Perceived Unfair Treatment Among US College Students Using Mobile Sensing: Pilot Machine Learning Study
Source: JMIR Form Res. 2025 Oct 31;9:e78657. doi: 10.2196/78657 (PMC12619020; doi:10.2196/78657)
Supplement: Multimedia Appendix 1 [file formative_v9i1e78657_app1.pdf]

EMA Questions

Table S1: Ecological Momentary Assessment (EMA) question and response options for self-reported unfair treatment.[a]

Component	Wording
**Question Stem**	"Did you experience unfair treatment for any of the following reasons [today/yesterday]?"
**Response Options**	Ancestry or National Origin<br>Gender<br>Sexual Orientation<br>Intelligence<br>Major<br>Learning Disability<br>Education or Income Level<br>Age<br>Religion<br>Physical Disability<br>Height<br>Weight<br>I was not treated unfairly

[a]The question stem was varied to ask about "today" in daily surveys and "yesterday" in twice-weekly surveys to ensure precise temporal alignment with passive sensor data. Participants could select any applicable reason or indicate they were not unfairly treated, as described in Sefidgar et al (2019).
