# Supplementary material for: Detecting Perceived Unfair Treatment Among US College Students Using Mobile Sensing: Pilot Machine Learning Study
Source: JMIR Form Res. 2025 Oct 31;9:e78657. doi: 10.2196/78657 (PMC12619020; doi:10.2196/78657)
Supplement: Multimedia Appendix 2 [file formative_v9i1e78657_app2.pdf]

Behavioral Feature Extraction Details

Activity Recognition Features

The Activity Recognition data stream uses the activity recognition APIs available on Android and iOS to infer and classify users' physical activities into predefined categories, such as walking, running, biking, being in a vehicle, or remaining still. For each time period, we compute the following features: (1) frequency of activity type changes; (2) total number of unique activity types; and (3) cumulative duration of all non-stationary activities (e.g., walking, running, biking). These features provide insights into the user's physical activity patterns.

Battery Features

We compute the total duration of all phone charging sessions within each time period based on the phone's battery charge status data. This feature provides insights into the user's phone usage patterns and charging habits.

Bluetooth Features

We extract Bluetooth features from scanned Bluetooth addresses recorded by the smartphone. These addresses are clustered into "self" (user's own devices), "related" (close contacts), and "others" (devices of other people). We then compute the following features: (1) the number of unique devices; (2) scans of the most and least frequently detected devices; and (3) the sum, average, and standard deviation of scans across all devices, as well as separately for "self" and "others" categories. These features capture the frequency and diversity of interactions with the user's own devices versus other people's devices, providing insights into social interactions and proximity-based communications.

Call Features

We extract call features including the number and duration of incoming, outgoing, and missed calls, as well as the total number of unique correspondents. These features, derived from the smartphone's call logs, provide insights into the user's communication patterns and call behavior.

Location Features

We extract location features from GPS data. This involves clustering samples to identify significant places visited by the user. For each time period, we compute the following features: (1) location variance, calculated as the sum of latitude and longitude variance; (2) total distance traveled; (3) average and variance of speed during movement; (4) circadian movement, indicating the extent of 24-hour cycle adherence; (5) location entropy, indicating the evenness of time distribution across significant places; (6) time spent at home, assuming the most frequented place during night hours (12am to 6am) as home location; and (7) clustering-based features, including the number of significant places, transitions, radius of gyration, time spent moving, time spent in rarely visited locations, and statistics (sum, maximum, minimum, average, and standard deviation) of length of stays at the top three significant places. These features capture various aspects of the user's spatial behavior and movement patterns.

Campus Map Features

We extract location features specific to campus environments. This involves categorizing different areas on a campus map into specific types, allowing us to label each location coordinate appropriately. The categories include dining areas, study areas, fraternity houses, residential/dorm spaces, exercise facilities, green spaces, and "other" areas (either off-campus or do not fit into any of the specified types). For each category, we extract the following features: (1) total time spent and percentage of time spent at the location type, relative to the length of the time period; (2) number of bouts, defined as continuous periods spent at the location type, including the total number of bouts and those lasting at least 10, 20, or 30 minutes; and (3) statistics (minimum, maximum, average, and standard deviation) of the lengths of these bouts. We also compute the total number of transitions between different location types to understand the user's mobility patterns and activity changes.

Additionally, we label specific teaching buildings and replicate several academic-related and multi-modal features introduced in the SmartGPA study: (1) class attendance features, including the number of classes attended and percentage of time spent in class; (2) study duration and study focus, by fusing location and activity recognition data; (3) party duration at fraternity houses; and (4) indoor mobility, measured by the duration of moving (walking or running) indoors; and (5) outdoor mobility, measured by the distance traveled outdoors.

Screen Features

We extract screen features from the smartphone's screen status (on, off, lock, unlock) data. Based on the definitions that: (1) a phone interaction bout is a continuous period during which the screen is on at the beginning and off/locked at the end; and (2) a screen unlock bout is a continuous period starting when the screen is unlocked and ending when it is locked again, we compute the following features: (1) frequency of screen unlocks per minute; (2) cumulative duration the screen is on; (3) cumulative duration the screen is in the unlocked state; (4) times of the day when the screen status first and last changes to on, unlocked and locked; and (5) statistics (maximum, minimum, average, and standard deviation) of the lengths of screen interaction and unlock bouts. These features provide insights into the user's phone usage patterns and interaction behaviors.

WiFi Features

We compute the total number of unique WiFi access points for each time period, defined differently across mobile operating systems: on Android devices, this count reflects the variety of WiFi networks detected through scanning, indicating the user's proximity to different networks; on iOS devices, it represents the number of networks to which the device has actively connected, offering insights into frequently visited locations. Overall, these features help in understanding the user's daily routines and social interactions.

Sleep Features

For this work, we focus on the main sleep episode of each day and use the following summary features from the Fitbit API. (1) start time of the sleep episode; (2) total sleep duration; (3) total time spent in different sleep states (asleep, awake, restless); (4) time in bed after waking up, and time in bed before falling asleep; and (5) sleep efficiency, calculated as the ratio of time asleep to total time in bed. These features provide insights into the user's sleep patterns and quality.

Steps Features

We extract steps features using the intra-day minute-by-minute activity data from the Fitbit API. A sedentary bout is defined as a continuous period during which the number of steps taken per minute is below a threshold (12 steps in our implementation), while an active bout is a continuous period with a per-minute step count at or above this threshold. Based on these definitions, we compute the following features: (1) total number of steps taken within the time period; (2) maximum number of steps taken per minute; (3) total number of active and sedentary bouts; (4) statistics (sum, maximum, minimum, average, and standard deviation) of the duration of active and sedentary bouts; and (5) maximum, minimum, and average step counts across all active bouts. These features provide insights into the user's physical activity, capturing both the intensity and frequency of movement throughout the day.
