# Supplementary material for: Detecting Perceived Unfair Treatment Among US College Students Using Mobile Sensing: Pilot Machine Learning Study
Source: JMIR Form Res. 2025 Oct 31;9:e78657. doi: 10.2196/78657 (PMC12619020; doi:10.2196/78657)
Supplement: Multimedia Appendix 3 [file formative_v9i1e78657_app3.pdf]

Demographic Variables Used in Baseline Classifier

The following table lists the demographic variables used to construct the demographic-only baseline classifier in user-independent modeling. All variables were self-reported and preprocessed according to standard machine learning practices (e.g., encoding categorical variables, etc.).

Table S2: Demographic variables used in constructing the baseline classifier.

Variable	Description	Value Type
age	Participant's current age	Continuous (years)
gender	Gender identity (e.g., male, female, non-binary, etc.)	Categorical
race	Self-identified racial group	One-hot encoded
ethnicity	Self-identified ethnic group	One-hot encoded
language	Language(s) spoken at home	One-hot encoded
handedness	Dominant hand	Categorical
generation	Generational status (e.g., immigrant, first generation, second generation)	Categorical
US_years	Number of years living in the United States	Continuous (years)
orientation	Sexual orientation (e.g., heterosexual, bisexual, homosexual, etc.)	Categorical
relationship	Relationship status (e.g., single, married, partnered, etc.)	Categorical
edu	Highest level of education completed (e.g., high school, some college, associate degree, etc.)	Categorical
college	Academic affiliation(s) based on college enrollment (e.g., College of Arts and Sciences, College of Engineering, School of Business, etc.)	One-hot encoded
student	Student subpopulation status (e.g., first-generation, fraternity/sorority member, international, commuter, athlete, disability, veteran)	One-hot encoded
employment	Employment status (e.g., part-time, full-time, unemployed, etc.)	Categorical
housing	Type of current housing (e.g., dormitory, apartment, off-campus, etc.)	Categorical
household_size	Number of household members by relationship type (e.g., spouse, children, parents, relatives, non-relatives)	Continuous (integer)
