# Supplementary material for: Detecting Perceived Unfair Treatment Among US College Students Using Mobile Sensing: Pilot Machine Learning Study
Source: JMIR Form Res. 2025 Oct 31;9:e78657. doi: 10.2196/78657 (PMC12619020; doi:10.2196/78657)
Supplement: Multimedia Appendix 4 [file formative_v9i1e78657_app4.pdf]

Comparison of rare event detection studies

Table S3: Summary of Rare Event Detection Studies

Study	Dataset Characteristics			Methodology			Results		
	**Domain**	**Size**	**Rarity**	**Model**	**Split**	**Baselines**	**Recall**	**Precision**	**F1**
Pillai et al (2023)	Mobile Sensing (Life Events)	10,106	1.9%	LSTM	80:10:10 (temp)	SVM, IF, LSTM	0.21	0.47	0.29
Coley et al (2023)	Healthcare (Suicide Risk)	~9.6M	0.2%	Random Forest	5-fold	None	0.53	0.09	0.16
Dong et al (2015)	Cellular (Crowd Events)	50,000	~6%	Non-ML	Unknown	External	0.92	0.07	0.13
Cheon et al (2009)	Environmental (Ozone States)	534	14%	Bayesian Net	Temporal	Decision Trees	0.58	0.49	0.53
Dangut et al (2022)	Mechanical (Aircraft)	1M+	1%	Deep RL	Unknown	RF, LSTM	0.85	-	-
Wong (2016)	Healthcare (Drug Mix-up)	227	21%	Logistic Regr.	70:30	None	0.529	0.644	0.575
**Our Work**	Mobile Sensing (Unfair Treatment)	5,085	3.73%	LSTM	90:10 (temp)	Uniform, Stratified	0.830	0.256	0.391
